# *pOsHAK1:OsSUT1* Promotes Sugar Transport and Enhances Drought Tolerance in Rice

**DOI:** 10.3390/ijms25042158

**Published:** 2024-02-10

**Authors:** Guang Chen, Wenli Lian, Anjing Geng, Yihan Wang, Minghao Liu, Yue Zhang, Xu Wang

**Affiliations:** 1 Institute of Quality Standard and Monitoring Technology for Agro-Products of Guangdong Academy of Agricultural Sciences, Guangzhou 510640, China; 2 Key Laboratory of Testing and Evaluation for Agro-Product Safety and Quality, Ministry of Agriculture and Rural Affairs, Guangzhou 510640, China; 3 Guangdong Provincial Key Laboratory of Quality & Safety Risk Assessment for Agro-Products, Guangzhou 510640, China

**Keywords:** rice, drought tolerance, sugar transport, inducible promoter

## Abstract

Plant cells accumulate osmotic substances (e.g., sugar) to protect cell components and maintain osmotic balance under drought stress conditions. Previous studies found that *pOsHAK1:OsFLN2* promotes sugar metabolism and improves the drought tolerance of rice plants under drought stress. This study further evaluated the effect of the ectopic expression of the *OsSUT1* gene driven by the *OsHAK1* promoter on the sugar transport and drought tolerance of rice. The results showed that the net photosynthetic rate and sucrose phosphate synthase activity of plants expressing the *OsSUT1* gene were not significantly different from those of wild-type (WT) rice plants under drought conditions. However, the sucrose transport rate in the phloem increased in the transgenic plants, and the sucrose contents were significantly lower in the leaves but significantly higher in the roots of transgenic plants than those in WT plants. The *pOsHAK1:OsSUT1* and *pOsHAK1:OsFLN2* transgenic lines had similar rates of long-distance sucrose transport and drought tolerance, which were higher than those of the WT plants. The relative water content of the transgenic plants was higher, while their water loss rate, hydrogen peroxide (H_2_O_2_), and malondialdehyde (MDA) contents were lower than those of the WT plants. The stress-responsive gene *OsbZIP23* and the antioxidant-related gene *OsCATB* were significantly upregulated in the drought-treated transgenic lines, while the senescence indicator gene *SGR* and the stress-responsive gene *OsNAC2* were down-regulated compared to WT plants. These results showed that promoting the long-distance sugar transport through the expression of *pOsHAK1:OsSUT1* could produce an improved drought tolerance effect similar to that of *pOsHAK1:OsFLN2,* providing an effective way to improve the drought tolerance of cereal crops at the seedling stage.

## 1. Introduction

Rice (*Oryza sativa* L.) is one of the most important food crops worldwide. Rice plants are mainly grown in irrigated and rain-fed lowland paddy systems since they consume more water than other cereal plants [1]. However, agricultural water is becoming increasingly scanty with the increasing climate changes and rapid industrialization and urbanization [2]. It is expected that by 2025, 15–20 million hectares of irrigated rice fields in Asia will experience continuous drought due to water shortage [3]. Therefore, to ensure food security, it is important to further understand the molecular response mechanisms of rice to drought stress, explore effective measures to improve rice drought tolerance, and develop new rice varieties resistant to drought [4].

Rice plants have developed several defense mechanisms to adapt to drought stress, among which accumulating small molecular organic substances serving as osmotic adjustment substances (such as amino acids, betaines, sugars, and organic acids) is one of the important protective strategies [5]. As a key osmoregulatory substance, soluble sugar enhances rice tolerance to drought by regulating osmotic balance, protecting protein structure, and maintaining membrane integrity [6]. Moreover, soluble sugars (mainly glucose and fructose) are re-transported from old leaves to young leaves to sustain the growth of new tissues under drought stress [7,8]. Drought affects the transportation and distribution of sugar in cellular and subcellular components by reducing the osmotic potential of cells [9]. Sugar transport is mediated by specific transporters, and there are three sugar transporter families in rice, i.e., monosaccharide transporters (MSTs), sucrose transporters (SUTs), and SWEETs [10]. These transporters are key determinants mediating the influx and efflux of various sugars under drought conditions [9]. *OsSUT1* is mainly expressed in the phloem cells of leaves and stamens of mature spikelets [11], which function in phloem loading [12] and participate in plant response to salt and drought stress [13].

Previous studies found that sugar metabolism is related to the drought stress responses of rice. The expression of *pOsHAK1:OsFLN2* (*pHAK1:FLN2*) under drought conditions promoted sugar metabolism in vivo and significantly improved the drought tolerance of rice at the seedling stage [14]. Sugar metabolism involves a series of physiological and biochemical processes, including carbon fixation, sucrose synthesis and loading, phloem transport, and sucrose unloading, decomposition, and utilization [15]. This study focused on the relationship between the long-distance transport of sugar and drought tolerance in rice, aiming to assess whether promoting sugar transport from the leaves (source organ) to the roots (sink organ) under drought conditions could improve the drought tolerance of rice. The promoter of *OsHAK1*, whose expression is induced and upregulated by drought/osmotic stress [16], was selected, and *OsSUT1*, involved in sucrose transport, was used as the functional gene. Two *pHAK1:FLN2* transgenic lines were used as the controls. The results showed that *pHAK1:SUT1* promoted sugar transport in drought-treated plants and significantly improved drought tolerance in rice, similar to *pHAK1:FLN2*. Therefore, promoting the long-distance transport of sugar could be an effective strategy for developing rice plants with high-drought tolerance.

## 2. Results

### 2.1. The Response of OsSUT1 to Drought Stress

**Figure 1:**
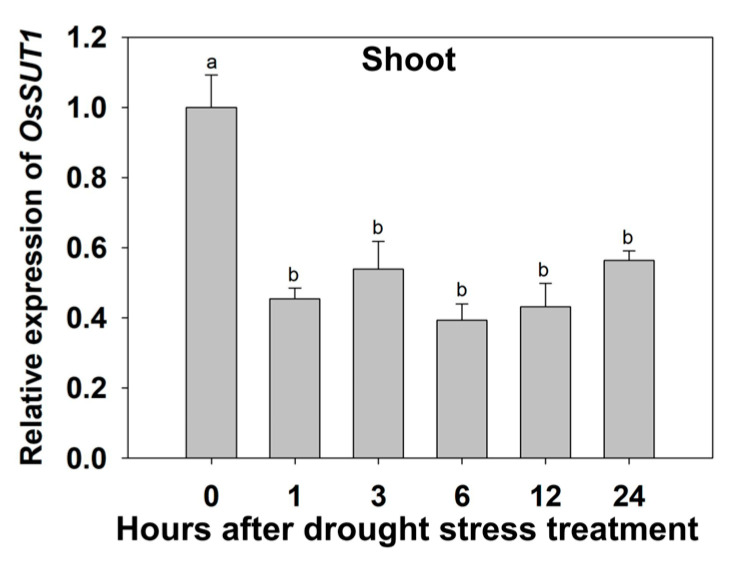
Effects of drought stress on the expression of *OsSUT1* in the shoots of wild-type rice plants. Transient expression of *OsSUT1* in the shoots of drought-treated wild-type rice cv Nipponbare. Rice seedlings were cultured in normal IRRI solution for 14 days and then transferred to a nutrient solution containing 20% polyethylene glycol (PEG) for different durations (0, 1, 3, 6, 12, and 24 h). The expression level at 0 h was set to 1. The values are mean ± standard error (SE) (*n* = 3). The different letters indicate significant differences at *p* < 0.05.

The qRT-PCR results showed that drought stress significantly inhibited the expression of *OsSUT1* in the shoots of rice plants (Figure 1). The *OsSUT1* transcript level decreased after 1 h of PEG (20%) treatment and reached the lowest point after 6 h but was maintained at 45–55% under normal conditions (Figure 1).

### 2.2. Generation of the pHAK1:SUT1 Transgenic Rice Lines

**Figure 2:**
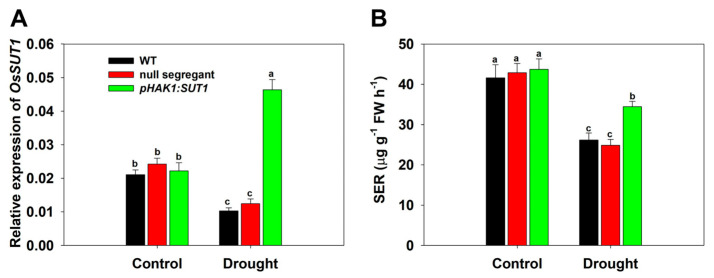
Comparison of *SUT1* expression and sucrose export rate between the T_1_ generations of the *pHAK1:SUT1* transgenic lines and wild type (WT) plants under normal and drought stress conditions. (**A**) The qRT-PCR analysis of the relative expression of *OsSUT1* in the shoots of rice seedlings. (**B**) Sucrose export rate (SER) in rice seedlings. The results of five isolates without the target fragment were combined to form a second control (null segregant), while those of five transgenic lines were combined to represent *pHAK1:SUT1.* The values are shown as mean ± standard error (SE). The different letters indicate significant differences at *p* < 0.05.

A differential analysis of drought responses was conducted between the *pHAK1:SUT1* transgenic lines and WT plants to examine whether promoting long-distance sugar transport can improve drought tolerance in rice. Positive plants were identified by β-glucuronidase (GUS) staining, and 21 independent transgenic lines were obtained in the T_0_ generation. Five positive lines of the T_1_ generation and their corresponding null segregants (NS) without the target segment were selected to analyze the expression level of *SUT1* and the sucrose export rate (SER). The NS and WT plants showed no significant difference under both normal and drought conditions; however, the expression of *SUT1* was inhibited in the shoots of WT and NS plants but upregulated by two folds in the transgenic plants under drought stress (Figure 2A). Meanwhile, the decline of the SER was significantly less pronounced in the transgenic lines than in the WT and NS plants (Figure 2B). Therefore, two homozygous plants of the T_2_ generation *pHAK1:SUT1* lines were selected for further analysis, and WT plants were used as the negative controls.

### 2.3. Effects of pHAK1:SUT1 on Rice Seedling Growth

**Figure 3:**
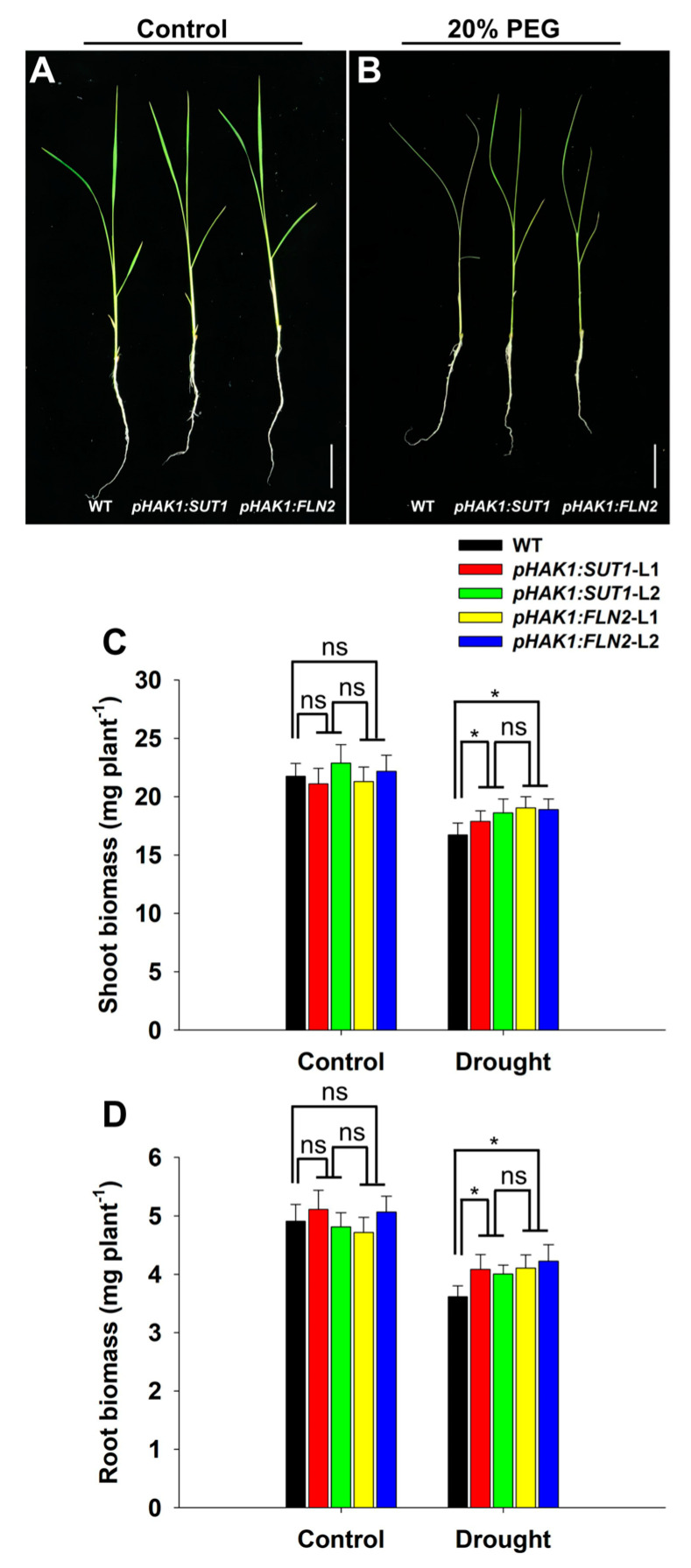
Growth comparison between the *pHAK1:SUT1* and *pHAK1:FLN2* transgenic lines and the wild type (WT) plants under normal and drought stress conditions at the seedling stage. (**A**,**B**) Growth performance of the seedlings under normal and 20% polyethylene glycol (PEG) treatment conditions. Bar = 5 cm. (**C**,**D**) Shoot (**C**) and root (**D**) biomass (dry weight). The values are mean ± standard error (SE) (*n* = 5). Significant differences between WT and the transgenic lines are indicated with asterisks (*p* < 0.05), and ns indicates non-significant differences at that significance level.

The WT and the T_2_ generations of *pHAK1:SUT1* and *pHAK1:FLN2* homozygous transgenic lines were cultured in normal and 20% PEG-containing nutrient solutions to evaluate the effect of *pHAK1:SUT1* expression on the growth and drought response of rice plants. Under normal culture conditions, the growth of transgenic plants was not significantly different from that of WT plants, with similar shoot and root biomass (Figure 3). However, the leaves of WT plants were severely wilted, while the dehydration effect was significantly reduced in the transgenic plants under drought stress. Drought inhibited the shoot and root growth of the *pHAK1:SUT1* and *pHAK1:FLN2* transgenic lines to a similar degree, and they were both significantly lower than those of WT plants. Consequently, the dry weight of the shoots of the PEG-treated *pHAK1:SUT1* and *pHAK1:FLN2* transgenic plants was 7–12% and 13–14% higher than that of WT plants (Figure 3C), while the dry weight of their roots was 11–13% and 14–17% higher than that of WT plants (Figure 3D).

### 2.4. Effects of pHAK1:SUT1 on Sugar Synthesis in Rice

**Figure 4:**
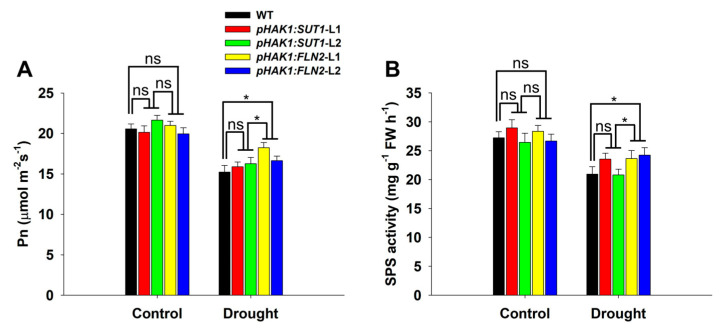
Sugar synthesis comparison between the *pHAK1:SUT1* and *pHAK1:FLN2* transgenic lines and the wild type (WT) plants under normal and drought stress conditions. (**A**) Net photosynthetic rate (Pn). (**B**) Sucrose phosphate synthase (SPS) activity. The values are mean ± standard error (SE) (*n* = 5). Significant differences between WT and the transgenic lines are indicated with asterisks (*p* < 0.05), and ns indicates non-significant differences at that significance level. FW, fresh weight.

The sugar synthesis levels in the leaves of the *pHAK1:SUT1* and *pHAK1:FLN2* transgenic plants were analyzed and compared with that of the WT plants to examine whether expressing *pHAK1:SUT1* improves the drought tolerance of rice by promoting sugar metabolism. Consistent with previous research results [14], the net photosynthetic rate (Pn) and sucrose phosphate synthase (SPS) activity of the *pHAK1:FLN2* transgenic plants were significantly higher than those of WT under PEG treatment (Figure 4), while the *pHAK1:SUT1* transgenic lines showed no significant difference from WT (Figure 4). This indicated that, unlike *pHAK1:FLN2*, *pHAK1:SUT1* expression did not alter sugar synthesis in rice under drought stress.

### 2.5. Effects of pHAK1:SUT1 on Sugar Transport in Rice

**Figure 5:**
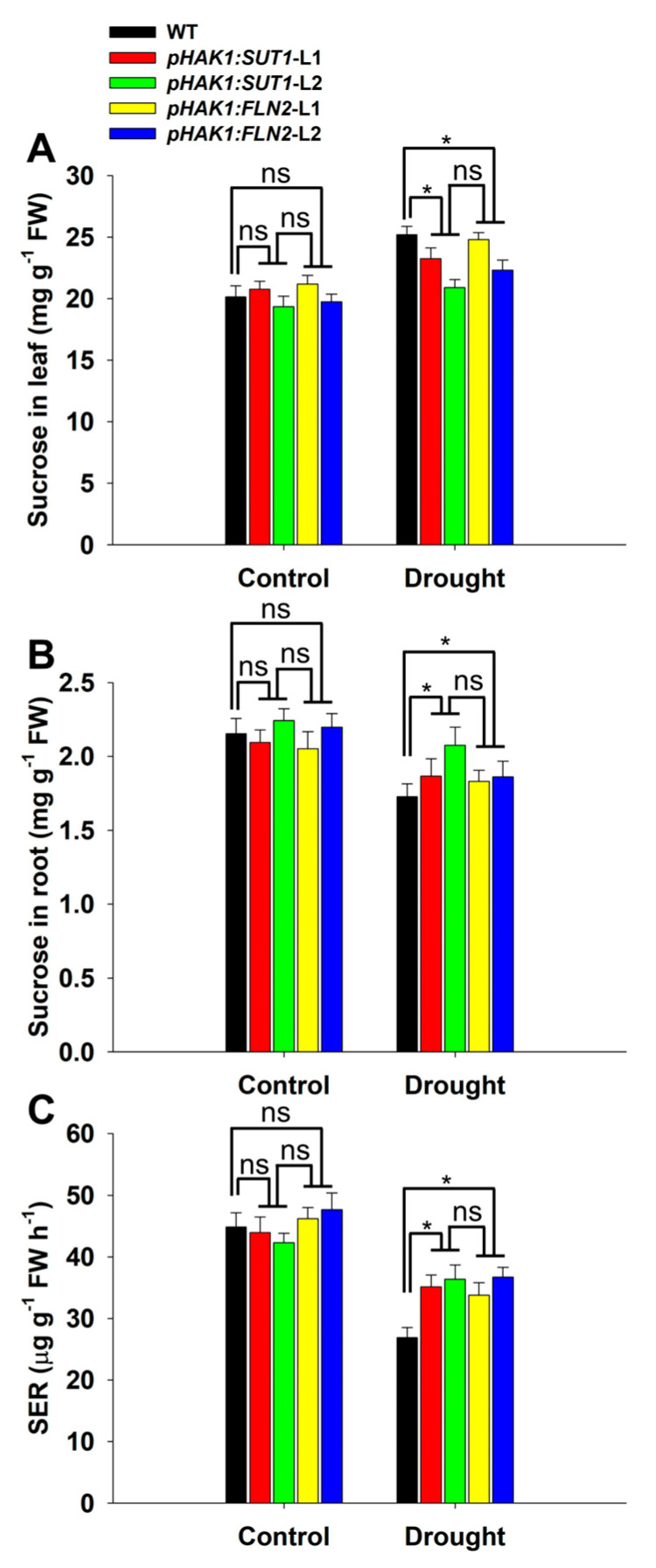
Sugar transport comparison between the *pHAK1:SUT1* and *pHAK1:FLN2* transgenic lines and the wild type (WT) plants under normal and drought stress conditions. (**A**,**B**) Sucrose contents of the leaves (**A**) and roots (**B**). (**C**) The sucrose export rate (SER) of the leaves. The values are mean ± standard error (SE) (*n* = 5). Significant differences between WT and the transgenic lines are indicated with asterisks (*p* < 0.05), and ns indicates non-significant differences at that significance level. FW, fresh weight.

The sugar transport levels of the *pHAK1:SUT1* and *pHAK1:FLN2* transgenic plants were analyzed and compared with that of the WT plants. The expression of *pHAK1:SUT1* and *pHAK1:FLN2* could significantly improve the long-distance transport of sugar under drought stress, as the SER values for the leaves of the *pHAK1:SUT1* and *pHAK1:FLN2* transgenic lines were 31–35% and 26–36% higher than that of WT, respectively (Figure 5C). However, the sucrose contents in the leaves and roots of the *pHAK1:SUT1* plants were 8–17% lower and 10–18% higher than that of WT, respectively (Figure 5A,B).

### 2.6. Effects of pHAK1:SUT1 on Total Root Length and Root Surface Area in Rice

**Figure 6:**
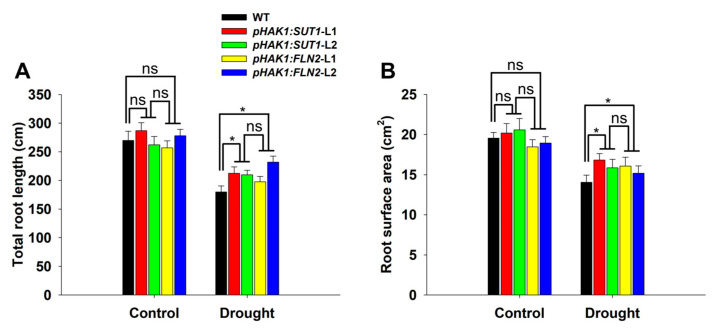
The performance of root growth comparison between the *pHAK1:SUT1* and *pHAK1:FLN2* transgenic lines and the wild type (WT) plants under normal and drought stress conditions. (**A**) Total root length. (**B**) Root surface area. The values are mean ± standard error (SE) (*n* = 5). Significant differences between WT and the transgenic lines are indicated with asterisks (*p* < 0.05), and ns indicates non-significant differences at that significance level.

The differences in the root response to drought stress between the *pHAK1:SUT1* and *pHAK1:FLN2* transgenic lines and the WT plants were analyzed. The PEG treatment significantly reduced the total root length and root surface area of all plants (Figure 6), but the effect was more pronounced on the roots of the WT plants. The roots of the *pHAK1:SUT1* and *pHAK1:FLN2* transgenic lines were inhibited to similar degrees, and they were both significantly lower than those of the WT plants. Consequently, the total root lengths of the drought-treated *pHAK1:SUT1* and *pHAK1:FLN2* plants were 16–19% and 11–27% higher than those of WT, respectively (Figure 6A). Similarly, the root surface areas of the drought-treated *pHAK1:SUT1* and *pHAK1:FLN2* plants were 13–20% and 9–14% higher than that of WT, respectively (Figure 6B).

### 2.7. Effects of pHAK1:SUT1 on Water Retention and Lipid Peroxidation in Rice

**Figure 7:**
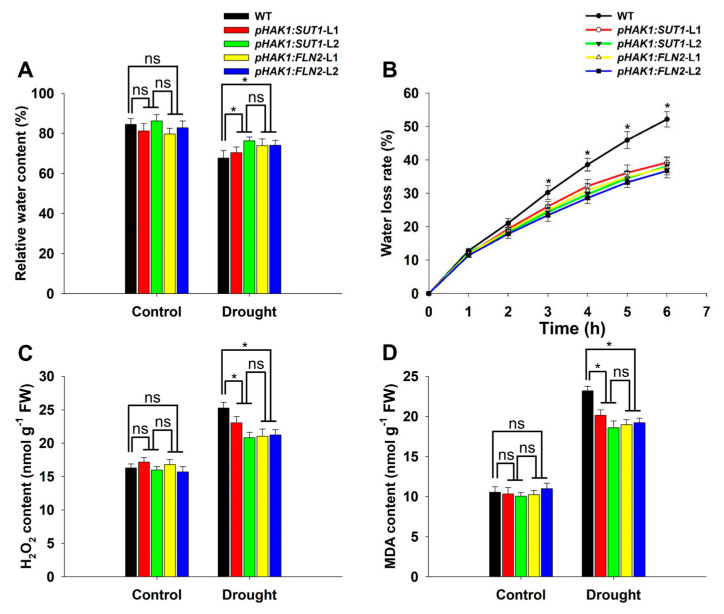
Water retention ability and lipid peroxidation of the *pHAK1:SUT1* and *pHAK1:FLN2* transgenic lines compared with wild-type (WT) plants under normal and drought stress conditions. (**A**) Relative water content. (**B**) Water loss rate. (**C**) Hydrogen peroxide (H_2_O_2_) content. (**D**) Malondialdehyde (MDA) content. The values are mean ± standard error (SE) (*n* = 5). Significant differences between WT and the transgenic lines are indicated with asterisks (*p* < 0.05), and ns indicates non-significant differences at that significance level. FW, fresh weight.

Compared with the plants under normal culture conditions, the relative water content (RWC) values of the drought-stressed WT and *pHAK1:SUT1* and *pHAK1:FLN2* transgenic plants decreased. The RWC values of *pHAK1:SUT1* and *pHAK1:FLN2* displayed no significant difference and were both significantly higher than that of WT (Figure 7A). This difference may be attributed to the different water loss rates of the plants. The detached leaves of the WT plants lost water at a higher rate than the transgenic lines (Figure 7B).

Under normal culture conditions, the WT and transgenic rice plants had similar H_2_O_2_ and MDA contents; however, after PEG treatment, the H_2_O_2_ contents in the *pHAK1:SUT1* and *pHAK1:FLN2* transgenic lines were 8–15% and 15–18% lower than that of WT, respectively (Figure 7C). The MDA contents of the *pHAK1:SUT1* and *pHAK1:FLN2* transgenic lines were 13–20% and 17–19% lower than that of WT, respectively (Figure 7D).

### 2.8. Effects of pHAK1:SUT1 on the Expression of Genes Related to Aging, Stress Response, and Anti-Oxidation

**Figure 8:**
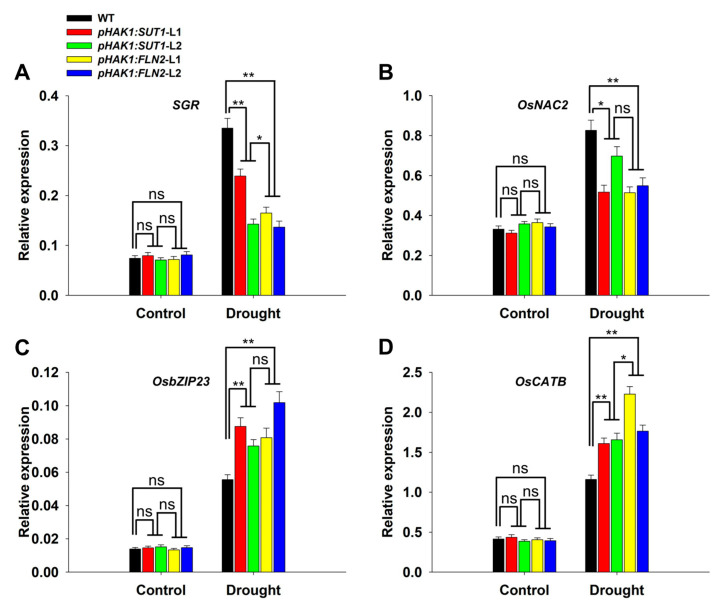
The relative expression levels of the senescence-associated, stress-responsive, and antioxidant-related genes in the *pHAK1:SUT1* and *pHAK1:FLN2* transgenic lines compared with those in the wild type (WT) plants under normal and drought stress conditions. The genes assayed were (**A**) *SGR*, (**B**) *OsNAC2*, (**C**) *OsbZIP23*, and (**D**) *OsCATB*. The values are mean ± standard error (SE) (*n* = 3). * and ** indicate significant differences (*p* < 0.05, <0.01) between the performance of the transgenic plants and that of WT plants. ns indicates no significant difference.

A differential analysis of gene expression was performed on normal and drought-stressed WT and *pHAK1:SUT1* and *pHAK1:FLN2* transgenic lines to further clarify the mechanism by which the expression of *pHAK1:SUT1* improves the drought tolerance of rice. The selected genes were divided into three categories: *SGR* (senescence indicator genes), *OsNAC2* and *OsbZIP23* (stress-responsive genes), and *OsCATB* (an antioxidant-related gene). The expressions of the selected genes were not significantly different between WT and the transgenic plants under normal conditions (Figure 8). Drought stress promoted the expression of all detected genes, but the degree of induction differed between transgenic and WT plants. Specifically, the expression levels of *SGR* in *pHAK1:SUT1* and *pHAK1:FLN2* plants were 30–57% and 50–59% lower than that of WT plants (Figure 8A), and the expressions of *OsNAC2* were 18–36% and 33–38% lower than that of WT plants, respectively (Figure 8B). Contrarily, the expressions of *OsbZIP23* in *pHAK1:SUT1* and *pHAK1:FLN2* plants were 1.36–1.58 and 1.46–1.83 times that in WT (Figure 8C), while those of *OsCATB* were 1.39–1.43 and 1.53–1.92 times that in WT, respectively (Figure 8D). These results indicated that negatively regulating the expression of the drought-induced senescence indicator gene *SGR* and the stress-responsive gene *OsNAC2* and positively regulating the expression of the stress-responsive gene *OsbZIP23* and the antioxidant-related gene *OsCATB* were one of the important reasons for the improvement of drought tolerance in the *pHAK1:SUT1* and *pHAK1:FLN2* transgenic lines.

## 3. Discussion

A previous study revealed that the drought tolerance of rice could be improved by promoting sugar metabolism under drought stress by transforming *pHAK1:FLN2* in rice [14]. Sugar metabolism involves complex physiological and biochemical processes, such as carbon fixation and sugar synthesis in source organs (e.g., leaves), and sugar decomposition and utilization in sink organs (e.g., roots), which also involve many molecular regulatory pathways [17]. Given the close relationship between environmental factors and the transport of sugar from “source” to “sink” [18], this study proposed a hypothetical strategy to improve the drought tolerance of rice by promoting the long-distance transport of sugar under drought stress. The promoter of the osmotic/drought response gene *OsHAK1* [16] was selected to drive the expression of the sugar-transporting gene *OsSUT1*, involved in the long-distance transport of assimilates in rice [11]. As expected, the *pHAK1:SUT1* transgenic lines grew normally under the control conditions without displaying adverse phenotypes (Figure 3). Only the sugar transport process rather than sugar synthesis was significantly changed in the *pHAK1:SUT1* transgenic plants compared with WT after PEG treatment (Figure 4 and Figure 5). These results suggest that, unlike *pHAK1:FLN2*, *pHAK1:SUT1* expression mainly affects the sugar transport in rice, which is closely related to the induced expression and biological function of *SUT1* under drought stress. Notably, such expression was consistently inherited by both T_1_ and T_2_ generations (Figure 2B and Figure 5C).

According to our comprehensive comparison analysis at the morphological, physiological, and molecular levels, the drought tolerance levels of the *pHAK1:SUT1* and *pHAK1:FLN2* transgenic plants were similar and significantly higher than those of the WT plants. The expression of *pHAK1:SUT1* and *pHAK1:FLN2* could alleviate the drought-induced inhibition of root growth, which was shown by the significantly higher root biomass, total root length, and root surface area of the transgenic plants than those of the WT plants under stress (Figure 3D and Figure 6). These can be explained by the sufficient supply of sucrose as a nutrient to the root system (Figure 5B). Root biomass and morphology are the key factors determining the ability of plants to obtain water and nutrients. Therefore, the optimized root growth performance was positively correlated with drought tolerance [19]. In this study, the PEG-treated *pHAK1:SUT1* and *pHAK1:FLN2* transgenic lines maintained a more developed root system, improving their stress tolerance at the morphological level.

Important physiological changes induced by *pHAK1:SUT1* and *pHAK1:FLN2* expressions were the increased content of osmotic regulators and the decreased accumulation of reactive oxygen species (ROS) (Figure 5B and Figure 7C). As an osmolyte, sugar responds to abiotic stress and helps plants resist stress [20]. The SER and sucrose contents in the roots of the *pHAK1:SUT1* and *pHAK1:FLN2* transgenic plants were similar and significantly higher than those of the WT plants (Figure 5B,C), which is beneficial for improved drought tolerance. In addition, drought stress induced excessive ROS accumulation in rice, damaging the membrane lipid structure [21]. MDA is the main product of lipid peroxidation of plant cell membranes, and its accumulation increases under drought stress [22]. In this study, the H_2_O_2_ and MDA contents of the *pHAK1:SUT1* and *pHAK1:FLN2* transgenic lines were significantly lower than those of the WT plants after PEG treatment (Figure 7C,D), indicating that the degree of drought-induced cell membrane damage was lower in the transgenic plants than that in the WT ones. Catalase (CAT, EC.1.11.1.6) is an important antioxidant enzyme, and plant CATs are typically encoded by three isozyme genes [23]. There are three isoenzyme genes in rice: *OsCATA*, *OsCATB*, and *OsCATC.* Water stress inhibited the expressions of *OsCATA* and *OsCATC* but significantly increased the expression of *OsCATB* [24]. Consistent with this report, our results showed that drought stress significantly upregulated *OsCATB* expression in the *pHAK1:SUT1* and *pHAK1:FLN2* transgenic lines compared to the WT plants (Figure 8D). Thus, the high expression of *OsCATB* may be one of the important mechanisms through which the PEG-treated transgenic plants alleviated drought stress-induced oxidative damage (Figure 7C,D).

Furthermore, qRT-PCR analysis revealed a correlation between gene expression differences and the drought-resistant phenotypes of the *pHAK1:SUT1* and *pHAK1:FLN2* transgenic plants. Drought stress causes leaf senescence. Several genes regulating the senescence of rice leaves have been identified, including *SGR*, *OsNYC1*, and *OsPAO* [25]. The senescence-induced gene *SGR* plays an important role in regulating chlorophyll degradation [26]. Overexpressing *SGR* caused oxidative stress and lesion-like cell death in rice seedlings [27], and it is thus used as a marker gene for leaf senescence. In this study, the *pHAK1:SUT1* and *pHAK1:FLN2* transgenic lines had similar patterns of drought stress-induced enhanced expressions of *SGR* and were both significantly lower than that of the WT plants (Figure 8A). This suggested that the expression of *pHAK1:SUT1* and *pHAK1:FLN2* can delay the drought stress-induced leaf senescence. Many studies have shown that the differential expression of stress/abscisic acid (ABA)-responsive genes enhances the tolerance of plants to various stresses [28,29]. OsNAC2 modulates ROS accumulation and negatively regulates the drought tolerance of rice by binding to the promoters of *OsAP37* and *OsCOX11* [30]. Drought stress induces the expression of *OsbZIP23*, which participates in the response of rice to drought stress by positively regulating *OsPP2C49* and *OsNCED4* and the ABA signaling and biosynthesis [31,32]. In this study, the expression level of *OsNAC2* in the *pHAK1:SUT1* transgenic plants was significantly lower than that in the WT plants under drought stress; however, the expression level of *OsbZIP23* was significantly higher in the *pHAK1:SUT1* transgenic plants than that in the WT plants under drought stress. These expression patterns were consistent with the expression profiles observed in *pHAK1:FLN2* (Figure 8B,C). This indicates that the differential expression of these two genes is another important contributor to the improved drought tolerance of the *pHAK1:SUT1* transgenic lines.

## 4. Materials and Methods

### 4.1. Materials

The full-length coding sequence (1836 bp) of the *OsSUT1* gene was amplified from the Nipponbare cDNA (accession number: AK100027) and ligated to the *pEASY*-Blunt cloning vector (Beijing Quanshijin Biotechnology Co., Ltd., Beijing, China) after purification. The promoter of *OsHAK1* (3037 bp upstream of the initiation codon) was amplified from the Nipponbare genomic DNA (accession number: AL606610) and was inserted into the *OsSUT1* cloning vector digested with *Bam*HI. The GBclonart seamless cloning kit (Suzhou Shenzhou Gene Co., Ltd., Suzhou, China) was used for the ligation process, and the obtained intermediate vector was named pHAK1-SUT1, which was subsequently used as the template to amplify the pHAK1-SUT1 fragment. After purification, the fragment was ligated to the pTCK303 vector digested with *Hind*III and *Spe*I, to obtain the final vector pTCK303-pHAK1-SUT1. The vector was electro-transformed into the *Agrobacterium* strain EHA105, and the transformed *Agrobacteria* were used to transfect Nipponbare calluses. The genetic transformation protocol described by Chen et al. [16] was adopted to create the *pHAK1:SUT1* transgenic rice lines. The *pHAK1:FLN2* transgenic line was constructed in our previous study [14].

The T_0_, T_1_, and T_2_ generations of the transgenic materials were planted in the transgenic plant nursery at the Guangzhou Dafeng Experimental Base of the Institute of Quality Standard and Monitoring Technology for Agro-products, Guangdong Academy of Agricultural Sciences. Three-week-old rice seedlings were treated for seven days with the hydroponic IRRI nutrient solution [33] supplemented with 20% (*w*/*v*) polyethylene glycol (PEG) 6000 (used to simulate drought stress). The phenotypic characteristics and various physiological and biochemical indices were recorded, and the gene expression levels were quantitatively analyzed. The experiment was conducted in an artificial climate chamber under a photoperiod of 14 h light (30 °C)/10 h dark (25 °C) and a relative humidity of around 70%. The nutrient solutions in all treatments were replaced every two days.

### 4.2. Method

#### 4.2.1. Determination of Pn

The Pn of rice leaves was measured from 9:00 a.m. to 11:00 a.m. with a Li-COR6400 portable photosynthetic instrument (Li-COR, Lincoln, NE, USA), as described by Li et al. [34].

#### 4.2.2. Determination of SPS Activity

After the drought treatment, the leaf samples were harvested and ground into powder in liquid nitrogen. The SPS activity was determined using the method described by Chen et al. [35] with an SPS kit (Suzhou Keming Biotechnology Co., Ltd., Suzhou, China).

#### 4.2.3. Determination of the SER

The ethylenediaminetetraacetic acid (EDTA) method described by Chen et al. [35] was adopted to measure the SER of the treated plants. Briefly, phloem exudate was obtained from the leaves by cutting the cut ends of the leaves and immediately immersing the leaves in 20 mL of EDTA solution (30 mM, pH = 7.0) for a 15 min incubation in the dark. The EDTA solution in the first round was discarded, and then the leaves were washed and transferred to fresh 10 mL of EDTA solution (30 mM) to avoid the influence of xylem exudate. The leaves were placed in a closed dark room with 75% relative humidity during the whole process. After 4 h, the sucrose concentration of the collected solution was measured using a sucrose kit (Suzhou Keming Biotechnology Co., Ltd.).

#### 4.2.4. Determination of Sucrose Content

After the stress treatment, the leaves and roots of the plants were harvested and ground into powder in liquid nitrogen. Sucrose was extracted with a sucrose kit and quantitatively determined using the method by Chen et al. [15].

#### 4.2.5. Real-Time PCR (qRT-PCR)

The steps described by Chen et al. [36] were used for the qRT-PCR analysis. RNA was extracted from the root and leaf samples of the wild type (WT) and transgenic lines under normal and drought conditions. The rice gene *UBQ5* (*LOC_Os01g22490*) was used as the reference gene, and the relative expression level was calculated as described by Li et al. [37]. The qRT-PCR primer sequences are shown in Table 1.

#### 4.2.6. Determination of Total Root Length and Root Surface Area

The root systems under different treatments were scanned with a root system analyzer (WinRhizoV4.0b, Regent Instrument Company, Quebec, QC, Canada), and the total root length and root surface area were recorded, as described by Song et al. [38]. Five individual plants were measured for each line in each treatment.

#### 4.2.7. Determination of the RWC and the Water Loss Rate

The RWC was determined using the method described by Zhao et al. [39]. Briefly, after the stress treatment, the leaves of the plants were detached and weighed, and their fresh weight (FW) was recorded. The leaves were then soaked in deionized water for 4 h, and the saturated weight (SW) was measured. After drying the leaves at 80 °C for 48 h, the dry weight (DW) was determined, and the RWC was calculated according to the following formula: RWC = (FW − DW)/(SW − DW) × 100%. The water loss rate of the detached leaves was determined using the method by Guo et al. [40]. Briefly, the leaves of WT and the transgenic plants at the seedling stage were cut and weighed and then exposed to the air at room temperature. The water loss rate was calculated after the leaves were weighed at the specified time points.

#### 4.2.8. Determination of H_2_O_2_ and MDA Contents

After the drought treatment, the leaves were harvested and ground into powder in liquid nitrogen. H_2_O_2_ and MDA were extracted with their corresponding kits (Suzhou Keming Biotechnology Co., Ltd.), and their contents were quantified using the method by Mostofa and Fujita (2013) [41].

#### 4.2.9. Statistical Analysis

The Tukey method was executed in IBM SPSS Statistics 25 software to analyze the significant differences between the lines and treatments. Different letters and asterisks (*) indicated significant differences at the *p* < 0.05 significance level, and “ns” indicated no significant differences.

## 5. Conclusions

Using the promoter of drought stress-induced gene *OsHAK1* to drive the expression of *OsSUT1* can promote the transport of sugar from source to sink under drought stress, reduce water loss rate and lipid peroxidation, and regulate the expression of senescence indicator genes, stress-responsive genes, and antioxidant-related genes to improve the drought tolerance of rice similarly to *pHAK1:FLN2*.

## Figures and Tables

**Table 1:** Primer sequences used for qRT-PCR assays.

Gene Name	Forward Primer (5′-3′)	Reverse Primer (5′-3′)
*UBQ5*	CTCGCCGACTACAACATCCA	TCTTGGGCTTGGTGTACGTCTT
*OsSUT1*	CGGTGACCCAAAGGGAACT	TGCCCTGACACCCTGGTT
*SGR*	GCAATGTCGCCAAATGACG	GCTCACCACACTCATTCCCTAAAG
*OsNAC2*	AAAAACAACCGCATTGGCAG	AGTCCTCATCTCCTCTGTCTAATCC
*OsbZIP23*	GGAGCAGCAAAAGAATGAGG	GGTCTTCAGCTTCACCATCC
*OsCATB*	GGTGGGTTGATGCTCTCTCA	ATTCCTCCTGGCCGATCTAC

## Data Availability

Data are contained within the article.
